# A study on the influencing factors of corporate digital transformation: empirical evidence from Chinese listed companies

**DOI:** 10.1038/s41598-024-56729-4

**Published:** 2024-03-15

**Authors:** Xu Zhao, Qi-an Chen, Haitao Zhang, Pengyu Chen, Shen Chen

**Affiliations:** 1 Surrey International Institute, Dongbei University of Finance and Economics, Dalian, 116025 Liaoning China; 2 School of Economics and Business Administration, Chongqing University (Campus B), Chongqing, 400030 China; 3 Cesar Ritz Colleges Switzerland, English Gruss Strasse 43, 3902 Brig, Switzerland

**Keywords:** Digital transformation, Influencing factors, Chinese listed companies, Socioeconomic scenarios, Sustainability, Human behaviour

## Abstract

In an era where digital technology is reshaping business landscapes, understanding the factors that drive corporate digital transformation is essential. In this paper we explore these influencing factors, focusing on Chinese A-share listed companies from 2007 to 2021. Our approach involved a comprehensive analysis of multiple variables through regression techniques to determine their impact on digital transformation. The findings reveal the drive for reform in the digital transformation endeavours of enterprises. Notably, companies with higher gearing, overhead, and accounts receivable ratios exhibit a stronger inclination towards digital transformation. Conversely, enterprises in monopolistic industries and those at the inception stage of their life cycle show less propensity for such transformation. The findings of this research not only shed light on the strategic decisions behind digital transformation in response to financial and competitive challenges but also provide actionable insights for policymakers and business strategists. This study underscores the importance of contextualizing digital transformation efforts within the unique framework of industry characteristics and company development phases.

## Introduction

At the dawn of the twenty-first century, a digital revolution, fuelled by rapid advancements in Internet technology, has swept across the globe, reshaping the socio-economic fabric and heralding a new era of industrial and technological transformation. This digital wave has not only sparked dynamic economic activities but also profoundly influenced the innovative locus of enterprises worldwide. Within this context, China’s digital economy has witnessed a revolution. According to the relevant data in the "2022 China Digital Economy White Paper", the scale of China's digital economy reached 45.5 trillion yuan by 2021, with a nominal growth of 16.2% year-on-year, higher than the nominal GDP growth rate of 3.4 percentage points in the same period, and accounting for 39.8% of the GDP, all of which manifest that the position of the digital economy in the national economy has become more stable and its supporting role more obvious. As a result, digital transformation has become a national development strategy, and the digital economy is developing at an unprecedented speed, with diverse and far-reaching impacts, which will bring profound changes to the production, life, and business behaviour of the current society. The Chinese government is proactively leading and encouraging the digitalization of small and medium-sized enterprises (SMEs), with supportive policies prompting businesses to embark on this transformation journey. For instance, Zhejiang Province, designated as a national pioneer in advancing the digital economy, has enthusiastically heeded the central government's directive. Large enterprises in the region are prioritizing innovation and growth, aiding smaller companies in their transition towards digitalization. As a result, the province has witnessed a continuous increase in its GDP, injecting dynamism into its economic expansion. Moreover, the Zhejiang provincial government offers assistance to enterprises seeking to transform, providing services such as talent development and vocational guidance^1^. It will become an important driving force that can reshape the core competitiveness of enterprises, improve the quality and efficiency of their operations, transform dynamic energy sources, and play a crucial role in leading China's economy to new economic growth points^2^.

However, the journey towards digital transformation is not without its challenges. The onset of the COVID-19 pandemic in 2020 laid bare the vulnerabilities of traditional business models, placing unprecedented pressure on companies striving for survival. In such a critical situation, the development of digital technologies and the digital economy has largely helped companies to reduce the risks associated with market uncertainty. Data from the National Development and Reform Commission^3^ illustrates this impact, showing substantial improvements in business and administrative efficiency through digital adoption. Yet, the digital transformation landscape is diverse and uneven across sectors. The results of the "Accenture 2021 China Digital Transformation Index"^4^, a survey of 398 companies, show that traditional retailers have consistent expectations for increased digital investment; technology-intensive industries such as electronics, high-tech, automotive, and construction have a high starting point when it comes to creating digital opportunities and are leading the way in digital transformation. In contrast, only 4% of companies in the chemical and building materials industries plan to increase their investment budgets for digital transformation in the next 1–2 years. The survey also reveals that even within the same industry different companies have different intentions for digital investment. For example, in the logistics industry, emerging courier companies are more willing to make digital investments because they have seen the benefits of digitalization in their past operations. The more traditional manpower-dependent transportation companies will be relatively less willing to invest in the face of digital transformation, and companies need to protect their capital by compressing investment costs. Such disparities necessitate an examination of the factors influencing digital transformation, taking into account the unique developmental trajectories and sector-specific challenges faced by enterprises.

Recognizing these complexities, in this paper we delve into an empirical exploration of the factors driving digital transformation in enterprises. Utilizing the Chinese capital market as our canvas, we examine A-share listed companies through corporate characteristics, governance, international openness, life cycle, and industry competition. China's A-shares include a wide range of industries, and during the time period selected for this study, a total of 84 industries of Chinese A-share companies were included in the statistics, 70 of which are included in this study (about 83.3% of total industries), as shown in the table below (Table 1).

**Table 1:** The A-Share industry distribution map.

	Industry	Counting	Proportion
1	Computer, communication and other electronic equipment manufacturing	949	11.4%
2	Pharmaceutical manufacturing	859	10.3%
3	Electrical machinery and equipment manufacturing industry	629	7.6%
4	Chemical raw materials and chemical products manufacturing industry	623	7.5%
5	Software and Information Technology Services	623	7.5%
6	Special equipment manufacturing industry	504	6.1%
7	General equipment manufacturing industry	374	4.5%
8	Automobile manufacturing	336	4.0%
9	Non-metallic mineral products industry	238	2.9%
10	Non-ferrous metal smelting and rolling processing industry	211	2.5%
11	Manufacturing of railway, ship, aerospace and other transportation equipment	164	2.0%
12	Fabricated metal products	163	2.0%
13	Rubber and plastic products industry	159	1.9%
14	Civil engineering and construction industry	152	1.8%
15	Wholesale trade	110	1.3%
16	Production and supply of electricity and heat	108	1.3%
17	Textile industry	108	1.3%
18	Agricultural and sideline food processing industry	106	1.3%
19	Wine, beverage and refined tea manufacturing	101	1.2%
20	Ferrous metal smelting and rolling processing industry	96	1.2%
21	Instrument manufacturing industry	96	1.2%
22	Textile and garment industry	93	1.1%
23	Food manufacturing	90	1.1%
24	Internet and related services	84	1.0%
25	Retail trade	84	1.0%
26	Paper and paper products industry	79	1.0%
27	Coal mining and washing industry	70	0.8%
28	Chemical fiber manufacturing industry	64	0.8%
29	Real estate	61	0.7%
30	Agriculture	59	0.7%
31	Mining support activities	58	0.7%
32	Professional and technical services	56	0.7%
33	Building decoration and other construction industry	53	0.6%
34	Non-ferrous metal mining and dressing industry	51	0.6%
35	Integrated	51	0.6%
36	Ecological protection and environmental management industry	49	0.6%
37	Telecommunications, radio and television, and satellite transmission services	44	0.5%
38	Business Services	44	0.5%
39	Other manufacturing industries	43	0.5%
40	Cultural, educational, artistic, sports and entertainment goods manufacturing industry	41	0.5%
41	Road Transport	38	0.5%
42	Press and publishing	35	0.4%
43	Petroleum processing, coking and nuclear fuel processing industry	32	0.4%
44	Printing and recording media reproduction industry	31	0.4%
45	Animal husbandry	26	0.3%
46	Timber processing and wood, bamboo, rattan, palm and grass products industry	25	0.3%
47	Furniture manufacturing industry	22	0.3%
48	Water production and supply industry	22	0.3%
49	Warehousing	19	0.2%
50	Water transport	18	0.2%
51	Hygiene	16	0.2%
52	Waste Resources Comprehensive Utilization Industry	14	0.2%
53	Gas production and supply industry	13	0.2%
54	Production of radio, television, film and sound recordings	11	0.1%
55	Leather, fur, feather and their products and footwear industry	11	0.1%
56	Postal Service	9	0.1%
57	Fishery	9	0.1%
58	Ferrous metal mining and dressing industry	8	< 0.1%
59	Forestry	8	< 0.1%
60	Culture and art industry	8	< 0.1%
61	Air Transport Industry	7	< 0.1%
62	Oil and gas extraction industry	7	< 0.1%
63	Catering industry	6	< 0.1%
64	Public Facilities Management Industry	6	< 0.1%
65	Education	6	< 0.1%
66	Agriculture, forestry, animal husbandry and fishery services	6	< 0.1%
67	Handling and transportation agency	6	< 0.1%
68	Railway transportation	3	< 0.1%
69	Research and experimental development	3	< 0.1%
70	Accommodation industry	2	< 0.1%
Total		8130	100.0%

In addition to this, China's A-share companies consist of three main sectors: private, state-owned, and foreign companies, all of which are covered in this study, with the relevant ratios as shown in the table below (Table 2).

**Table 2:** Property ownership structure diagram of a-share companies.

EquityNature	Counting	Proportion
Private	4854	58.41%
State-owned	3136	37.74%
Foreign	229	2.75%
Others	91	1.10%
Total	8310	100.0%

The aim of this research is to offer actionable insights for companies navigating the digital era, guiding them in refining their digital strategies to effectively harness the opportunities and tackle the challenges presented by the digital economy. Furthermore, through our findings, we aim to contribute valuable policy recommendations, supporting the sustainable and healthy growth of China's real economy in this digital epoch.

## Literature review

### The meaning of enterprise digital transformation

At the core of digital transformation is the use of digital technologies to improve the existing organizational model of business management, fill the "data gaps" between different departments of the company, and enable the redesign of production and operation structures and management models, thus improving the efficiency of resource allocation and innovating management models^5^. For traditional companies, digital transformation means integrating digital technologies into different aspects of production and operations. This is evident in the case of China Huashi Enterprises Co., Ltd., which incorporated the "Shanjian Cloud" digital platform into its construction processes, fundamentally altering its approach to project management and operational efficiency.

The amplifying effect of digital technologies on economic development is leveraged to adapt the company's vision, strategy, organizational structure, capabilities, culture, and processes to the rapidly changing digital environment, as demonstrated by Xuzhou Construction Machinery Group Co. Ltd. (XCMG). Through its digital transformation, initiated with an SAP ERP project in 2009, XCMG has evolved into a digital platform serving external enterprises^6^, showcasing a complete change into a digital enterprise^7^.

Digital transformation is a systematic process to improve the spatio-temporal distribution of technology, capital, human resources, and materials through efficient data flows. This process also mitigates the impact of environmental uncertainty on the enterprise, as seen in the broader trend of Chinese companies embracing digital transformation by deploying cloud computing (CC), artificial intelligence (AI), and big data (BD)^8^.

The digital transformation process is characterized by "uninterrupted" and "break-and-build" innovation, which requires companies to move away from traditional business management models. This shift places higher demands on changes in organizational structure and input movement forms^9^. It follows that the TOE theoretical framework equally applies to the study of which factors influence the adoption and the advancement of digital transformation in firms. On this basis, in this paper we combine existing research results and the actual situation in China. We focus on variables under two levels, specifically organizational and environmental, to further explore how they affect the degree of digital transformation of firms. More specifically, at the corporate level, the main focus is on internal factors, i.e., firm characteristics and degree of internationalization; at the environmental level, the emphasis is on the role of industry competition from the external perspective.

### Drivers of digital transformation

The drivers of digital transformation can be understood as the reasons why companies undergo digital transformation, and the study of these drivers can help companies better carry out digital transformation by enabling them to understand the difficulties they face when undergoing such transformation. Hence, the study of digital transformation drivers is a very important topic, and scholars have studied both internal and external drivers.

In their analysis of the internal drivers of digital transformation of enterprises, Bhattacharya et al.^10^ argues that enterprises in different life cycles have different levels of willingness to engage in digital transformation, and therefore see the life of the enterprise as one of the internal drivers of enterprises' digital transformation. Zhang^11^ believes that the main reason companies want to undergo digital transformation is because they want to better respond to market demand and are committed to reduce their production costs and increase productivity. Globally renowned enterprises, such as Microsoft and Alibaba, have made use of digital technology to carry out digital and intelligent transformation in all aspects of enterprise management, production, operation, and marketing, thereby facilitating the rapid development of enterprises. Wang and Chen^12^ found that in the face of the expanding scale of enterprises, a lot of time and energy are wasted in the process of information transfer between departments, and digital transformation can effectively solve the problem of poor information transfer, so the initial purpose of digital transformation for many enterprises is to solve the problem of information transfer between internal organizations. For example, Schneider Electric opened up the digital technology and data silos scattered all over the enterprise, integrated the dispersed digital technology with its own operation technology, enabled the digital technology to empower the operation technology, and gradually realised digital connectivity and data interconnection in the aspects of enterprise production and operation, management, and business^13^.

In their analysis of the external drivers of enterprise digital transformation, Zhao^14^ points out that along with the development of shared technologies, which has played a driving role in the innovation of enterprise digital transformation, the deployment of new generation shared technologies is the key to the digital transformation of enterprises. Wang^15^ considered the impact of specific environmental factors on enterprise digital transformation and found that in the face of the COVID-19 pandemic, under the influence of changing market demand, enterprises that carry out digital transformation can better face the complex market situation, so during the pandemic many enterprises accelerated the pace of digital transformation. In 2020, Yintai Department Store became a department store "in the cloud", allowing consumers to "shop in the cloud" through the Meow Street App and Taobao Live Streaming Room, and making itself the world's first 24-h "new retail" department store, the first "new retail" department store in the world to stay open 24 h a day. Thanks to its early digital transformation layout, even in 2020, when the pandemic hit hard, the sales performance of Yintai Department Store showed a counter-trend growth^16^. This shows that changes in the market environment can be the main driving force for digital transformation.

In summary, we believe that the drivers of enterprise digital transformation can be divided into two aspects, which are the internal drivers and the external drivers of enterprises; external drivers include the needs of market competition, the push-back mechanism of clients and the leading role of technological development, while internal drivers encompass the life cycle of the enterprise, the financial situation, and the organizational situation of the enterprise.

## Methods

### Theoretical mechanisms

#### The TOE framework and its application

The TOE (Technology, Organization, Environment) framework, introduced by Tornatzky and Fleischer in 1990, serves as a fundamental model that offers a comprehensive perspective for examining how firms adopt innovative technologies. It categorizes the influencing factors into three critical dimensions: technology, organization, and environment. Technological factors refer to existing technologies and technologies that have not yet been introduced into the market by the enterprise^17^; organisational factors refer to some organisational characteristics related to resource utilisation and adoption, including organisational size and scope, characteristics of the management structure, and other relevant resources available within the organisation^18^; and environmental factors refer to the macro-environment in which the enterprise is situated, including the governmental environment, the macro-environment, the industry environment, and the intensity of competition^19^. This framework has been widely applied in research to explore the complex dynamics of technological innovation and adoption across various industries and geographical regions.

For instance, Oliveira and Martins^20^ utilized the TOE framework to delve into ERP adoption in European firms, revealing the critical roles of technological benefits, organizational readiness, and the competitive environment in facilitating adoption. Another Lin^21^ application in Taiwan's industries examined cloud computing adoption, pointing out key drivers such as technological compatibility, organizational agility, and the regulatory environment. These studies highlight the TOE framework's capability to provide valuable methodological insights and identify relevant factors that drive or hinder technology adoption in different contexts.

The choice to employ the TOE framework for investigating digital transformation in Chinese-listed companies is supported by its proven adaptability and depth of analysis in these examples. Despite critiques regarding its potential oversimplification of the adoption process and the challenge of capturing the whole socio-technical interplay, the successful application of the TOE framework in the studies showcases how careful operationalization of its dimensions and integration with other theoretical perspectives can yield insightful conclusions. Therefore, this study aims to adapt and extend the TOE framework to the context of digital transformation among Chinese listed companies.

#### Corporate governance theory

Corporate governance theory, originating in the United Kingdom and rapidly developed in the United States, has branched into various research directions, with "shareholder primacy theory" and "stakeholder theory" being the most recognized. The shareholder primacy theory views the maximization of shareholders' interests as the primary purpose of a business. This theory posits that shareholders, as the company's owners, promote a stable "capital-employed labor" governance model, emphasizing the importance of focusing on shareholder value as a guiding principle for corporate governance^22^. In this model, corporate governance mechanisms are designed to ensure that managers act in the best interests of shareholders, thereby aligning management actions with shareholder goals.

In contrast, stakeholder theory, diverging from shareholder primacy, thinks that the company is a responsible entity whose operational goals should encompass not only shareholder interests but also social responsibility and stability^23^. This perspective broadens the scope of corporate governance by advocating for considering a more comprehensive range of interests beyond those of shareholders alone. According to Chen and Zheng^24^, stakeholder theory asserts that stakeholders' involvement impacts company development, enabling them to share in the firm's control and influence governance decisions. This implies a governance framework that integrates diverse interests, promoting balanced decision-making that accounts for the needs of all stakeholders.

An organization's equity structure and ownership can measure the corporate governance dimension. With the emergence of the information economy, principal-agent theory has gained prominence, advocating for the separation of ownership and management in companies. This theory, as described by Shi, Connelly, and Hoskisson^25^, clarifies the relationship between principals (shareholders) and agents (management). It highlights the governance challenges associated with this separation, including potential conflicts of interest between owners and managers. The principals elect a board of directors who, in turn, appoint management to make operational decisions, establishing a governance mechanism to oversee management actions and ensure they align with shareholder interests.

Li and Li^26^ suggest that the management expense ratio serves as a metric for principal-agent costs, capturing the extent of agency costs, such as inappropriate managerial consumption and on-the-job expenses. This ratio is pivotal in assessing the effectiveness and efficiency of corporate governance, particularly in principal-agent dynamics. It provides an empirical measure to evaluate how well the governance structure mitigates agency problems, ensuring that management acts in the shareholders' best interests while balancing other stakeholders' needs.

The role of corporate governance theory in this context is to provide a conceptual framework for understanding and addressing the complexities of managing and governing corporations. By delineating the rights, roles, and responsibilities of different actors within the corporate structure, these theories help craft governance mechanisms that aim to reduce conflicts, enhance accountability, and promote sustainable business practices. In the digital transformation era, these governance theories are particularly relevant as they offer insights into how companies can navigate innovation and change challenges, ensuring that governance structures support strategic objectives while maintaining a commitment to ethical standards and stakeholder engagement.

### Factors influencing the digital transformation and research hypothesis

Among the existing studies, there is a rich discussion around the role of enterprise digital transformation outcomes, whereas there is still some room for exploring the areas of their driving mechanisms and influencing factors. Currently, domestic scholars have conducted in-depth studies from the perspectives of transformation strategy^27^, audit^28^, and social responsibility^29^ based on the digital transformation practices of Chinese enterprises, while the exploration of the influencing factors of enterprise digital transformation is relatively underdeveloped and rarely approached from the economic and technological perspectives, which is not conducive to the complete revelation of the logic of corporate decision-making in the digital economy era, and cannot clarify the behavioural mechanism of important corporate strategies. Although the central government of China has issued a series of construction standards and improved the top-level design for the digital development of the real economy, the digital transformation of enterprises is still affected by various factors such as enterprise characteristics, corporate governance, internationalization (degree of openness), life cycle, and industry competition due to their different actual operating conditions.

In order to better analyse the influencing factors of enterprise digital transformation, in this paper we start from the organizational level under the TOE framework, and conduct research from three perspectives: enterprise characteristics, corporate governance, and life cycle. More specifically: enterprise characteristics are further refined into six specific variables: enterprise size, capital structure, accounts receivable ratio, management expense ratio, profitability, and revenue share of overseas business; based on corporate governance theory, two variables, equity structure and ownership, are selected; in terms of life cycle, enterprise life cycle and age are taken as two variables; at the environmental level of TOE, the focus is on the impact of industry competition, so the selection of the Herfindahl–Hirschman Index (HHI) index as a variable.

#### Enterprise size

From the perspective of enterprises, promoting digital transformation requires a large amount of capital for technology investment, which also generates a huge scale of sunk costs. Digital transformation requires the support of the latest digital technologies such as Big Data (BD), Cloud Computing (CC), Artificial Intelligence (AI), and Blockchain, which will inevitably generate a large amount of investment in research and development, and only sufficient funds can guarantee the stable operation of enterprises in their digital transformation. Compared with small and medium-sized enterprises (SMEs), large enterprises have larger capital and richer resources, and their own information technology expertise is relatively high, so they have more advantages in digital transformation. In addition, because digital transformation involves more digital technology supply, and has certain requirements for enterprise-related infrastructure, enterprises need to have higher risk tolerance to be able to quickly complete digital transformation. SMEs already have the natural disadvantages of small scale and weak risk resistance, and with their generally single business model and limited financing channels, they will have difficulty coping with the huge cost pressure brought about by digital transformation in the opposite direction. At the same time, digital transformation requires large investments in market development and raw material procurement, and specialized networks, equipment, and information systems as the infrastructure for transformation. However, since most SMEs lack awareness of digital transformation, the internal digital infrastructure of these enterprises is often not sound enough, which means that SMEs need to spend a significant amount of money for the procurement of related equipment and facilities to improve digital transformation, which will certainly increase the cost of digital transformation of SMEs, and even seriously affect their security and possibly even survival. The larger enterprises, on the other hand, can recognize the necessity of digital transformation due to their understanding of the basic principles of digital transformation in the process of daily development, and their own capabilities and resources are sufficient to support digital transformation.

Bai et al.^30^ empirically concludes that at the organisational level, firm size, board independence and availability of finance have a significantly positive impact on digital transformation of firms, suggesting that expanding the size of the firm is conducive to facilitating digital transformation of firms. Based on this, we propose:

H1: The size of an enterprise is proportional to its degree of digital transformation.

#### Capital structure

The financing problem has been an important obstacle to business development and reducing the cost of capital has been considered as a business development goal; according to Wen et al.^31^, the increase in business risk leads to additional financing needs and high leverage, which generates more interest costs. Moreover, external investors, especially bank investors, are aware of such problems, and in the face of high risk, will inevitably demand higher returns that match the potential risk, which will inevitably increase the financing costs of firms. It follows that highly leveraged firms will have higher financing costs than existing non-highly leveraged firms. Digital transformation can improve information processing capabilities and market expectations, further optimizing and improving firms' investment and financing behaviour, laying the foundation for lower financing costs.

First, digital transformation reduces information asymmetry, reinforces positive market expectations, and reduces financing costs. The strengths of digitally transformed organizations in the areas of information acquisition, storage, transmission, and identification significantly improve their ability to process large amounts of information internally and externally. On the one hand, companies can use their established information data experience to improve their operations and gain insight into market needs through data processing. On the other hand, Che, Duan, and Wang^32^ argue that companies that process and export information effectively can "market" this information to gain more support from external investors. This two-way access to information significantly reduces information asymmetry and has a more positive effect on highly leveraged firms, helping to increase the availability of capital and reduce liquidity constraints. Since investors have a better understanding of the company, they can demand a lower "risk premium" from the company, thus reducing the cost of financing.

Second, digital transformation has a positive effect on the creditworthiness of highly indebted companies and can improve their investment and financing behaviour to a certain extent and reduce financial bottlenecks. With digital transformation, companies are able to allocate resources more efficiently and are able to improve access to capital, which can significantly reduce the financial risk of highly indebted companies and reduce their need to wean themselves off financial risk. With the impact of digital transformation, the potential for business growth can be further stimulated, managers can allocate capital more wisely, and companies can improve the efficiency of their operations in pursuit of higher returns. Recognizing this higher potential, external investors lower their expectations of risk, which in turn lowers their financing costs.

In the process of digital transformation, through the close integration of all aspects of business management and digital technology, can effectively reduce costs, improve efficiency and increase profits, therefore, the pursuit of production efficiency, economic profits and market position of the enterprise, more likely to digital transformation^33^; Zhang^11^ research found that to meet the market demand for small and medium-sized enterprises is the primary driver of the digital transformation, and substantially cost reduction and productivity improvement are the other two important driving factors. Based on this, we propose:

H2: There is a significant positive effect of corporate indebtedness on corporate digital transformation, and the higher the indebtedness, the more willing the company is to undergo digital transformation.

#### Percentage of accounts receivable

As an important component of a company's working capital, accounts receivable have a direct impact on a company's ability to realize funds and the speed of cash flow^34^. Accounts receivable are an indispensable component of an entity's liquidity, and can help companies gain more customers in the face of fierce market competition. However, the effective management of accounts receivable is also a difficult task for companies, which can increase the workload of accounting processing; companies also need to face the pressure when collecting accounts receivable. Once accounts receivable are deferred and not effectively collected, this will seriously affect the liquidity of the enterprise and increase the operating expenses and cost of capital; secondly, the existence of accounts receivable inevitably exaggerates part of the company's operating performance, and even if an allowance for doubtful accounts is established, the risk of potential loss still exists; finally, accounts receivable do not have cash flow corresponding to operating profit, and the related costs must be provided by capital, which increases the risk of impaired liquidity and leads to an increase in corporate financial risk.

With digital transformation, companies are able to manage their receivables in a networked and digital way, which can significantly improve the quality of their receivables management. This is mainly due to the fact that under the influence of digitalization, enterprises are able to assess customer quality more accurately, reducing the risk of bad debts and improving the efficiency of their capital investments. The perfection of accounts receivable analysis systems cannot be achieved without comprehensive data support. Digital transformation of enterprises can improve the efficiency of data processing and therefore can promote the increase of accounts receivable turnover. Compared with traditional offline channels, online platforms have more stable revenue and shorter repayment cycles, and therefore higher accounts receivable turnover rates. This not only reduces the risk of bad debts, but also improves capital turnover and operational efficiency. Gregory^35^ found that with the emergence of the concept of digital transformation, a series of industrial analyses claimed that digital transformation improves firm performance; and Hu^36^ confirmed that digital transformation of firms has a positive contribution to firm performance. Based on this, we propose:

H3: The accounts receivable ratio is directly proportional to the degree of digital transformation, and the higher the ratio, the more willing the company is to undergo digital transformation.

#### Management overhead ratio

Digital transformation can promote information sharing among enterprises, which in turn can help them improve management efficiency. According to Liu, Bai, and Dong^37^, new-generation digital technologies such as BD, blockchain, and CC are breaking down the "data silos" of enterprises, enabling the sharing of enterprise information, accelerating the "disintermediation" of enterprise internal governance, integrating and rationalizing management methods, and achieving the effect of cutting management costs and improving management efficiency. Digital transformation enables enterprises to create an intelligent, rational, and efficient information management model for operation and management, thereby improving work efficiency. At the same time, digital transformation allows the enhancement of enterprise information technology management, which helps break the business barriers between departments, reduce information asymmetry between departments in the enterprise, eliminate barriers to information transmission, improve timeliness, enhance the efficiency of enterprise operations, and reduce administrative costs. Digital transformation helps to increase the awareness of information management among business managers, which in turn improves management efficiency, and can support the development and application of digital technologies by increasing the resources and energy invested in the business^27^. Firms can achieve better production management, reduce their market transaction costs such as those for search, information, transportation, delivery, and management, promote better organization and resource allocation, improve supply chain management capabilities, and enhance value creation through data aggregation, data analysis, and data-driven decision-making^38,39^. In an experiment with listed companies, Li et al.^39^ found that the digitization of companies themselves could effectively improve the efficiency of internal management. Digital transformation enables management to manage human resources more effectively, while the use of systematic and intelligent contracts can avoid cost problems, contract disputes, and fraud issues, thus reducing risks and management costs and improving management efficiency. Based on this, we propose:

H4: The overhead ratio is directly proportional to the degree of digital transformation, and the higher the expense ratio, the more willing the digital transformation is.

#### Profitability (ROA)

The pursuit of increased profitability is the main reason for companies to make the change and transformation. The transformation of a company involves a great cost, and if the benefits gained after the transformation are not project to match the costs paid, then the incentive to transform the company will be greatly affected. Digital transformation of an enterprise means new costs, increased training costs and an uncertain future for the enterprise. Significant investments in building teams of experts, acquiring new hardware and software, developing and designing business products, establishing digital channels, and redesigning intelligent processes are all elements of a successful digital transformation. However, it is clear that these changes are not entirely due to digital technologies and that increased investments do not necessarily lead to increased production. From the initial investment phase (mainly investments in hardware, software, and training services) to the mid-term internalization phase (training and adaptation of the hardware, software, and services, integration into existing enterprise management systems, etc.) and subsequent value creation, digital transformation requires a very complex and time-consuming process for a company to be able to finally achieve the goal of increasing its profitability. Digital transformation will inevitably change the original organizational structure of enterprises, change their management process and operation model, and affect the whole enterprise management system. As a consequence, companies with relatively strong profitability will be open to digital transformation, but they also tend to maintain the current management model, will not readily try risky operations, and will have a more conservative attitude when facing digital transformation. Based on this, we propose:

H5: Profitability has no significant effect on the willingness of companies to undergo digital transformation.

#### Revenue share of overseas business

With the implementation of "going global", China's Foreign Direct Investment (FDI) has grown rapidly and has become one of the world's largest investors. Following the "One Belt, One Road" strategy, more and more Chinese companies are venturing into foreign markets and doing business abroad^40^. Less attention has been paid in the existing literature to the issue of how the share of overseas business affects digital transformation, and different scholars hold different views on this issue. Stallkamp, Hunt, and Schotter^41^ argue that companies with a high share of overseas operations are more willing and motivated to undertake digital transformation, and companies with a high degree of internationalization have a broader perspective and are more likely to be exposed to international cutting-edge ideas. In addition, the digital economy can promote technological and industrial innovation and upgrading, which can lead to efficient use of resources and thus optimize the efficiency of resource use, which also encourages companies with a high degree of internationalization to undergo digital transformation.

Another view is that companies with a high degree of internationalization are not more willing to undergo digital transformation. Wu and Tian^42^ suggest that companies with a high proportion of overseas operations have more barriers to digital transformation, which affects their willingness to engage in digital transformation and the extent to which they do so. It is more difficult to obtain real-time data and other information between business units based in and outside of China. At the same time, countries have imposed various controls on the flow of cross-border data to ensure information security, resulting in the restricted flow of such data. In recent years, the Personal Information Protection Law, the Data Security Law, and other laws and regulations on data management have been promulgated one after another, and the management of international information transmission has become stricter. Furthermore, due to the geographical dispersion and differences in business environments, the offshore operations of multinational enterprises are often highly heterogeneous, making it difficult to achieve integrated domestic and foreign operations^41^. Consequently, the degree of internationalization may affect the unified coordination of domestic and overseas resources in the process of digital transformation of enterprises, and thus the degree of digital transformation of enterprises. Zhan and Ouyang^43^ argue that in addition to objective-level constraints, the weak motivation of some enterprises with a high proportion of overseas business to carry out reform is due to the fact that such enterprises are generally dependent on upstream enterprises and have a stable revenue stream. This makes them lack the willingness and motivation for digital transformation. However, it is easy to find that the above views are based on theoretical level analysis and lack empirical support. Based on this, in this paper we aim to explore the relationship between digital transformation and internationalization of enterprises from both theoretical and empirical levels, and propose the following hypotheses:

H6: A high percentage of overseas revenue has an inverse effect on the digital transformation of enterprises, and enterprises with a high percentage of overseas revenue have a lower willingness to engage in digital transformation.

For ease of reading, the following table summarises the six assumptions about firm characteristics at the organisational level (i.e., 'O' in the TOE theoretical framework) (Table 3).

**Table 3:** Recap of H1-H6.

Enterprise characteristics	Hypotheses
Enterprise size	H1: The size of an enterprise is proportional to its degree of digital transformation
Capital structure	H2: There is a significant positive effect of corporate indebtedness on corporate digital transformation, and the higher the indebtedness, the more willing the company is to undergo digital transformation
Percentage of accounts receivable	H3: The accounts receivable ratio is directly proportional to the degree of digital transformation, and the higher the ratio, the more willing the company is to undergo digital transformation
Management overhead ratio	H4: The overhead ratio is directly proportional to the degree of digital transformation, and the higher the expense ratio, the more willing the digital transformation is
Profitability (ROA)	H5: Profitability has no significant effect on the willingness of companies to undergo digital transformation
Revenue share of overseas business	H6: A high percentage of overseas revenue has an inverse effect on the digital transformation of enterprises, and enterprises with a high percentage of overseas revenue have a lower willingness to engage in digital transformation

#### Shareholding structure

The impact of equity structure factors on the digital transformation of firms has been extensively explored in the existing literature. On the one hand, some scholars argue that the higher the concentration of equity in a company, the easier it may be to make production and management decisions^44^; on the other hand, there is also literature that shows that excessive concentration of equity may lead to "one voice" that can affect the optimal transformation of a company^45,46^.

Mao et al.^45^ find empirically that shareholders are more inclined to participate in the decision-making process of digital transformation than in other business decisions because of the more far-reaching impact of digital transformation strategy on the company. This leads to more arbitrariness and difficulty to change corporate decisions, which affects the decision of digital transformation. Therefore, Vial^46^ states that the willingness of companies to digitally transform is more pronounced in companies with less concentrated shareholdings than in companies with relatively concentrated shareholdings.

Yin et al.^44^ assert that companies with a concentrated shareholding have a greater sense of "big ownership" when making business decisions, which encourages them to actively exercise their supervisory power and thus facilitates the efficient implementation of decisions. Gul, Kim, and Qiu^47^ contend that high equity concentration can bring about a certain degree of monitoring and control, which is more pivotal in markets with poor external corporate governance mechanisms. This is because in the absence of external management constraints, shareholders are forced to actively participate in supervisory management, which can only be effective if ownership is concentrated^48^. Similarly, controlling shareholders who own a large percentage of shares have a strong incentive to actively constrain and influence the actual power of management. This helps mitigate agency problems and thus facilitates decision-making^49^. In summary, we propose:

H7: Corporate shareholding structure plays an inverse effect on digital transformation; the more concentrated the shareholding structure, the lower the willingness of firms to transform digitally.

#### Ownership

The ownership type of a firm represents the organizational system, background, and resource environment of the firm, and firms with different ownership types have different corporate goals, which in turn influence their investment decision preferences^49^. Chinese enterprises can be divided into state-owned (SOEs) and non-state-owned (mostly private and a few foreign-owned) based on the nature of ownership (property ownership). In studies of ownership and digital transformation of firms, scholars have mostly discussed state-owned enterprises in terms of their operations and the personal characteristics of their executives^50,51^.

SOEs have relatively fixed business models and business philosophies, which are not easily influenced by the market and can take their place in the market with their unique advantages^51^. This leads to a lack of incentive for SOEs to innovate in the process of digital transformation. In contrast, non-SOEs, which are in a fierce and uncertain market environment, need to conform to the digital technology trends in the new era in order to create competitive advantages in future development. At the same time, digital transformation fits the innovation needs of non-SOEs to create advantages, thus driving them to invest more in innovation activities for digital transformation^52^.

In terms of corporate executives, Porfírio et al.^53^ noted that SOEs are subject to stricter government regulation than non-SOEs, and executive appointments and decisions are subject to government interference. The selection and promotion of SOE executives is based on managerial competence and political considerations^54^, which hinders the full development of their managerial capabilities and makes them more willing to stay in their comfort zone rather than risk innovative transformation. In addition, executive compensation in SOEs is subject to the government's Executive Compensation Regulations, which reduces the incentive for executives to initiate digital transformation^52^. Based on this, we propose:

H8: SOEs have a lower willingness to undertake digital transformation compared to private enterprises.

The 2 hypotheses proposed under the corporate governance theory at the organisational level (i.e., "O" in the TOE theoretical framework) are presented in the following table (Table 4).

**Table 4:** Recap of H7 and H8.

Enterprise characteristics	Hypotheses
Shareholding structure	H7: Corporate shareholding structure has an inverse effect on digital transformation; the more concentrated the shareholding structure, the lower the willingness of firms to transform digitally
Ownership	H8: SOEs have a lower willingness to undertake digital transformation compared to private enterprises

#### Enterprise life cycle

According to Gort and Kleppe's^55^ enterprise life cycle theory, an enterprise is like a living organism, and its growth until death is called a life cycle. In different life cycle stages, enterprises' own business characteristics, resource reserves, and financing channels differ greatly, and also encounter different degrees of agency and information asymmetry problems. As a result, there are also some differences in the willingness of enterprises to engage in digital transformation in each cycle. Digital transformation is a major strategic decision, which makes it important for companies to avoid blind digital-related investments in the process of digital transformation. Several scholars have studied the impact of digital transformation on firm performance across different life cycles and found that its role differs across the life cycle^10,56^. The same differences may exist across time in terms of the impact of the life cycle a firm is in on its willingness to digitally transform, which needs to be verified empirically.

Chanias, Myers, and Hess^57^ argue that the willingness to transform digitally increases as the business development cycle progresses. Miller and Friesen^58^ suggest that start-ups are young and do not have sufficient resources and experience to support a large-scale transformation. For companies in the growth stage, development focuses on gradual stabilization and rapid sales growth and resource accumulation, trying to achieve advantage accumulation to help the company gain greater scale. Therefore, companies in the growth stage have the motivation to carry out digital transformation to achieve cumulative development. Companies in the maturity period have a stable and mature business model and organizational structure, relatively rich and stable profits, and a strong willingness to innovate on their own^48^. The mature structure and strong willingness and ability to innovate enable mature enterprises to have higher willingness to face digital transformation. In addition, mature enterprises tend to accumulate certain digital resources and capital in their development, so mature enterprises have a certain foundation for digital transformation^59^.

Finally, Bhattacharya, Chang, and Li^60^ argue that digital transformation can bring new dynamism to declining firms and new ways to improve firm performance. Therefore, companies in recession also have some willingness to transform digitally. However, some scholars point out that in the majority of enterprises in recession, even if they are willing to carry out digital transformation, few of them can really implement it, due to objective capacity constraints and lack of funds^61^. In summary, in this paper we propose the following hypothesis:

H9: Firms in the growth and maturity stages of the enterprise life cycle have a stronger willingness to engage in digital transformation.

#### Business age

Yin^44^ empirically verified the effect of firm's year of establishment (Age) on willingness to engage in digital transformation. The results demonstrate that the more experienced the firm is in operation, the more motivated it is to undergo digital transformation. Wu et al.^62^ pointed out that longer-established companies with more operational experience are more likely to have sufficient capabilities and resources to effectively support the digital transformation reform process, make full use of information advantages, and achieve scale effects; at the same time, the large amount of resources within the company can promote the synergistic development of the company through digital technology, thus improving its innovation capability. Therefore, companies with more operational experience have a higher willingness to engage in digital transformation.

Companies with operational experience undergo digital transformation with the aim of using digital technologies to enhance and innovate existing products^63^, and explore and develop new, potentially disruptive business models to remain competitive and generate new revenues^11,64^. In contrast, companies that are still young and lack operational experience have a relatively low willingness to undergo digital transformation because they differ from experienced companies in managing their operational focus. Meanwhile, Strange, Chen, and Fleury^61^ point out that the management capacity and cost required to conduct digital transformation are huge challenges for most start-ups. Therefore, start-ups have low motivation to undertake digital transformation due to lack of experience, capacity, and cost constraints. In summary, we propose the following hypothesis:

H10: Enterprise age has a positive effect on digital transformation willingness; the earlier an enterprise is established, the more experienced it is in operation, and the more willing it is to undergo digital transformation.

The 2 assumptions made by considering the life cycle of the firm at the organisational level (i.e., "O" in the TOE theoretical framework) are summarised in the table below (Table 5).

**Table 5:** Recap of H9 and H10.

Enterprise characteristics	Hypotheses
Enterprise life cycle	H9: Firms in the growth and maturity stages of the enterprise life cycle have a stronger willingness to engage in digital transformation
Business age	H10: Enterprise age has a positive effect on digital transformation willingness; the earlier an enterprise is established, the more experienced it is in operation, and the more willing it is to undergo digital transformation

#### Herfindahl–Hirschman Index (HHI)

The Herfindahl–Hirschman Index (HHI) measures market concentration and competition among firms within an industry^65^. It is calculated by summing the squares of the market share percentages held by all firms within the market, offering insights into the competitive dynamics and the concentration level in an industry^65^. A higher HHI indicates a higher level of market concentration, suggesting fewer competitors and potentially less competition. In contrast, a lower HHI reflects a more fragmented market with more competitors and higher levels of competition.

HHI is a tool that measures the level of competition among firms. The degree of industry competition reflects the intensity of competition among firms with limited resources, and Nasiri^66^ states that within industries with low levels of industry competition, firms are less exposed to competitors' interference with their resources and access to financing, making them less motivated to create competitive advantage through digital transformation reforms. Conversely, the higher the level of competition within an industry, the more motivated firms are to make digital transformation reforms, expecting to take advantage of the digital dividend and promote competition in the industry. Some scholars have pointed out that the competitive environment and market changes are the key factors and main drivers for digital transformation from the internal and external perspectives of companies^8,67^. The evolution of consumer demands and industry technologies can drive companies to use digital platforms and technologies to find creative solutions. Changes in the external environment can have an impact on organizational behaviour and structure, which in turn can have an effect on the propensity to adopt technology in the digital transformation of companies. Firms with low HHI face a more difficult competitive environment and have a higher willingness to undergo digital transformation. At the same time, some scholars argue that firms with low HHI are more willing to digitally transform because they recognize that digital technologies bring disruptions to the markets in which they operate^40^. They facilitate the recombination of existing products and services to generate new forms of digital offerings^68^, lower barriers to entry, and hinder the sustainability of the competitive advantage of existing players^69^. For example, online platforms enable the redefinition of existing markets by facilitating the exchange of digital goods and services^70^.

In contrast, Nasiri^66^ notes that firms with high HHI operate in a business with high barriers to entry and a stable flow of benefits. Such companies have little or no need to quickly differentiate themselves in a homogenous competitive market. Digital technology can help enterprises quickly grasp market development trends through calculation and analysis, find insightful problems, and make timely optimization and rectification in the rapidly changing market environment. Therefore, the willingness of enterprises with high HHI to carry out digital transformation is low. Therefore, we propose the following hypothesis:

H11: The higher the HHI, the lower the willingness of enterprises to engage in digital transformation.

In other words, the hypotheses that emerge from the environmental dimension of the TOE theoretical framework (i.e., the "E" in TOE) are the following (Table 6).

**Table 6:** Recap of H11.

Enterprise characteristics	Hypotheses
Herfindahl–Hirschman Index (HHI)	H11: The higher the HHI, the lower the willingness of enterprises to engage in digital transformation

### Data, variables, and model setting

#### Data sources

In this study we utilized data from A-share listed enterprises spanning from 2007 to 2021 as the research subjects, based on a combination of data availability and reliability. The variables for the degree of digital transformation of listed enterprises and the Herfindahl–Hirschman Index (HHI) of industry concentration were acquired from the China Stock Market & Accounting Research Database (CSMAR), while all other financial data were obtained from the Wind Financial Terminal (WIND). To ensure the validity of the results, several measures were taken to screen and purify the data, including: (1) exclusion of the financial industry sample to account for differences in accounting standards and industry-specific characteristics; (2) elimination of companies with abnormal listing statuses, such as ST and PT, to prevent abnormalities in their operations from affecting the regression results; and (3) exclusion of observed samples with significant missing data and removal of the first and last 1% of extreme outliers to eliminate their interference. These steps were taken to ensure the reliability of the research results.

In this study we use data collected over a long period of time, from 2007 to 2021, as the research sample, consisting of a diverse range of enterprises, including those in various sectors and industries. This broad sample allows for a comprehensive examination of the digital transformation of enterprises, including those that have undergone the transformation and those that have not yet done so at the time of observation. This approach reduces the potential for selective bias and increases the reliability of the regression results by avoiding the restriction of the research scope to only those enterprises that have undergone digital transformation.

#### Selection of variables and model setting

The aim of the present study is to investigate the relationship between digital transformation and financial performance, with the degree of digital transformation serving as the explanatory variable. This concept has been extensively explored in both the business community and academia. As suggested by Qi and Xiao^71^, the digital transformation of enterprises is primarily underpinned by the integration of AI, blockchain, CC, and BD technologies into the everyday operations of the enterprise. This digital transformation not only enhances the underlying technologies but also empowers the enterprise's production, management, and sales through digital technology applications. To measure the degree of digital transformation, in this study we draw on previous research^72^ and use the annual reports of the sample enterprises. The reports are analysed for the frequency of occurrence of terms such as "AI technology", "blockchain technology", "CC technology", "BD technology", and "digital technology application". To avoid heteroscedasticity, the combined number of occurrences of these five terms is logarithmised, and 1 is added. In a robustness test, the "digital technology application" term is excluded and the study focuses on the basic digital technology level, including AI, blockchain, CC, and BD technologies. The frequency of occurrence of these four terms is increased by 1, de-logarithmised, and used as replacement variables in the robustness test. According to Wu et al.^73^, based on the annual reports of A-share listed enterprises, we use the text recognition function of Python crawler to measure the level of digital transformation of enterprises by using the method of keyword "searching-matching-totaling" in this paper.

To determine the impacting factors on the level of digital transformation in enterprises, in this paper we select several variables at both the enterprise and industry levels as explanatory variables. Subsequently, we carry out regression analysis to investigate the correlation between these variables and the degree of digital transformation:

In this paper, we gauge the size of the enterprise by taking the natural logarithm of its total assets. We believe that larger companies are more inclined and pressured to embrace digital transformation due to the significant financial commitment it entails. Moreover, the ability to undergo digital transformation varies among enterprises of different sizes.

To evaluate the capital structure of a firm, we look at its gearing ratio. Capital availability plays a critical role in determining the level of digital transformation a company can undertake, and the gearing ratio offers insights into a company's capacity to secure external funding and manage its debt effectively.

We measure receivable structure by examining current accounts receivable as a percentage of total assets. Companies with a higher proportion of accounts receivable may be more motivated to engage in digital transformation, which can optimize accounts receivable management, enhance business distribution, and improve capital turnover efficiency.

The profitability of a company is evaluated using the return on total assets. Highly profitable companies typically have a strong financial position and are better positioned to take on the risks associated with digital transformation. However, they may also be more cautious and resistant to altering their business strategies, potentially leading to reluctance in embracing digital transformation.

Management costs are analyzed by looking at a company's overhead ratio as a percentage of operating income. Companies with higher management costs are incentivized to reduce day-to-day expenses through digital transformation, which can improve overall management efficiency.

The ownership concentration within a company is determined by the percentage of shares held by its largest shareholder. While a concentrated shareholding structure may aid decision-making in digital transformation, concerns have been raised regarding the authoritarian nature of management in such cases. Therefore, the influence of shareholding structure on digital transformation effectiveness requires further investigation. Moreover, a dummy variable is employed to distinguish between state-owned and private enterprises and evaluate their readiness for digital transformation.

The level of international openness of a business, indicated by the ratio of overseas revenue to total revenue, serves as a proxy for assessing its degree of global engagement. Generally, a higher proportion of overseas revenue correlates with a greater level of internationalization within the enterprise.

This study combines Dickinson's^74^ cash flow-based classification with Gort and Kleppe's^55^ five-stage life cycle model, comprising introduction, growth, maturity, obsolescence, and decline stages. The research concentrates on variations in digital transformation readiness across companies at different life cycle phases. Companies in the introductory and growth stages may prioritize steady expansion and adhere to a conservative business approach. A dummy variable is introduced to represent the company's life cycle; a value of 1 denotes companies in growth and introduction phases, while a value of 0 signifies those in maturity, obsolescence, and decline stages, allowing for an examination of digital transformation readiness across various life cycle stages.

While business life cycle theory evaluates a company's operational, investing, and financing activities based on cash flows, it offers limited insights into the company's managerial expertise. Conversely, a company's longevity and business experience exhibit a stronger correlation with its willingness to embrace digital transformation. This study employs the establishment years of a company as a proxy for business experience in empirical analysis.

Industry competition is measured using the Herfindahl index as a proxy variable. Companies are classified based on the 2012 SEC Industry Classification Code, and the Herfindahl index is computed utilizing individual companies' operating revenue shares to gauge market concentration. A higher coefficient signifies a more monopolistic industry, while a lower coefficient indicates higher competition. Unlike competitive markets, companies in monopolistic industries may lack a strong incentive to pursue digital transformation, as they can generate substantial profits without emphasizing operational enhancements (Table 7).

**Table 7:** Explanatory Table of Variables.

Variable type	Variable code	Variable name
Explained variables	${\text{ln}}\_digi2$	The extent of enterprises' digital transformation
Explanatory variables	$lev$	Capital structure
	$size$	Asset size
	$REC$	Receivable structure
	$ROA$	Profitability
	$Mfee$	Management costs
	$Top1$	Shareholding structure
	$SOE$	Corporate properties
	$overseainc$	Degree of openness
	$ecycle$	Business life cycle
	$Firmage$	Business operation
	${hhi\_d}$	Industry competition

In summary, combined with the relevant theory in this paper, the empirical model is set up as follows:

$${{\text{ln}}\_digi2}_{it}={b}_{1}{size}_{it}+{b}_{2}{lev}_{it}+{b}_{3}{REC}_{it}+{b}_{4}{ROA}_{it}+{b}_{5}{Mfee}_{it}+{b}_{6}{Top1}_{it}+{b}_{7}{SOE}_{it}+{b}_{8}{overseainc}_{it}+{b}_{9}{ecycle}_{it}+{b}_{10}{Firmage}_{it}+{b}_{11}{hhi\_d}_{jt}+{u}_{i}+{v}_{t}+\in$$

where i represents the observed sample firms, t represents the year of observation, and j represents the industry to which the firm belongs. u_i represents individual fixed effects, v_t represents time-fixed effects, and ∈ is the residual of the model fit. STATA, EXCEL, and SPSS software were used for data operations and analysis.

## Empirical results and analysis

### Descriptive statistics

Before performing the baseline regression, we conducted descriptive statistics on the variables used in the study to comprehend the overall distribution of the sample. The mean and standard deviation of the core explanatory variable ln_digi2, which measures the extent of digital transformation, were 1.1911 and 1.3617, respectively. The minimum and maximum values of this variable were 0 and 6.3008, indicating a wide range of digital transformation among the sample enterprises. Some enterprises had not undergone digital transformation, while others varied in their extent of transformation (Table 8).

**Table 8:** Descriptive statistics results.

Variables	(1)	(2)	(3)	(4)	(5)
	N	mean	sd	min	max
Size	37,994	22.1053	1.4101	10.8422	29.9477
Lev	37,994	0.4630	1.5862	− 0.1947	178.3455
ROA	37,994	0.0422	0.3782	− 64.8192	20.7876
REC	37,994	0.1156	0.1043	0.0000	0.9750
Top1	37,994	0.3479	0.1515	0.0029	0.8999
SOE	37,994	0.3779	0.4849	0.0000	1.0000
FirmAge	37,994	2.8367	0.3784	0.0000	4.1589
Mfee	37,994	0.3789	23.2718	− 0.7569	3,404.6113
ln_digi	37,994	0.7553	1.1787	0.0000	6.2596
overseainc	37,994	0.1258	0.2098	− 0.5142	1.3186
hhi_d	37,994	0.1071	0.1286	0.0142	1.0000
ln_digi2	37,994	1.1911	1.3617	0.0000	6.3008
ecycle	37,994	0.9394	0.2385	0.0000	1.0000
Number of code	4383	4383	4383	4383	4383

The other explanatory variables exhibit similar characteristics and highlight the fact that, despite all the companies being listed on the A-share market, there are significant variations in their financial, operational, and managerial conditions. This further validates the representativeness of the research sample. Therefore, a comprehensive description of all the variables is not required in this study.

### Multicollinearity test

To prevent the presence of multicollinearity in the regression results, the variance inflation factor (VIF) test was applied to eliminate multicollinearity among the explanatory variables (Table 9).

**Table 9:** VIF Test.

Variables	VIF value	Variables	VIF value
SOE	1.21	Size	1.21
Top1	1.12	Lev	1.10
Firmage	1.09	REC	1.07
ROA	1.06	Mfee	1.05
Overseainc	1.03	Hhi_d	1.03
Ecycle	1.03	Mean vif	1.09

The results of the variance inflation factor test revealed that the mean value of each explanatory variable and the model was below the threshold of 10, which is considered the indicator for the presence of multicollinearity. Therefore, it can be concluded that there is no multicollinearity among the variables selected for this study, and the baseline regression analysis can proceed.

### Baseline regression

In this section, factors that were previously theorized to impact a firm's willingness to undergo digital transformation will be tested empirically. To ensure the validity of the results, a double fixed effects model that accounts for individual and time-level omitted variables will be employed in the empirical regressions. The outcomes of the baseline regressions are presented below (Table 10).

**Table 10:** Baseline regression results.

	(1) ln_digi2	(2) ln_digi2	(3) ln_digi2	(4) ln_digi2	(5) ln_digi2	Corresponding hypothesis	
Size	0.2338*** (31.28)	0.2406*** (32.19)	0.2450*** (32.71)	0.2448*** (32.44)	0.2463*** (32.64)	H1	Accept
Lev	0.0083*** (2.91)	0.0094*** (3.28)	0.0094*** (3.32)	0.0092***	0.0096*** (3.37)	H2	Accept
				(3.22)			
REC	0.7302*** (9.74)	0.7318*** (9.80)	0.7469*** (9.99)	0.7424*** (9.93)	0.7404*** (9.90)	H3	Accept
ROA	− 0.0016 (− 0.08)	0.0045 (0.22)	0.0025 (0.12)	0.0035 (0.17)	0.0051 (0.25)	H5	Accept
Mfee	0.0003* (1.93)	0.0003* (1.67)	0.0003* (1.68)	0.0003* (1.71)	0.0003* (1.71)	H4	Accept
Top1		− 0.6669*** (− 11.65)	− 0.6636*** (− 11.60)	− 0.6312*** (− 10.89)	− 0.6246*** (− 10.78)	H7	Accept
SOE		− 0.0335 (− 1.38)	− 0.0385 (− 1.59)	− 0.0414* (− 1.70)	− 0.0408* (− 1.68)	H8	Accept
overseainc			− 0.2543*** (− 6.42)	− 0.2529*** (− 6.38)	− 0.2548*** (− 6.43)	H6	Accept
ecycle				− 0.0300* (− 1.78)	− 0.0291* (− 1.73)	H9	Accept
FirmAge				0.1450*** (3.25)	0.1178*** (2.63)	H10	Accept
hhi_d					− 0.3177*** (− 6.20)	H11	Accept
_cons	− 4.6814*** (− 29.50)	− 4.5628***	− 4.6216*** (− 29.13)	− 4.9323*** (− 26.68)	− 4.8630*** (− 26.26)		
		(− 28.81)					
Year	Yes	Yes	Yes	Yes	Yes		
*N*	37,823	37,823	37,823	37,823	37,823		
adj. *R*^2^	0.292	0.296	0.298	0.298	0.298		
F	1,055.2357	968.8880	928.9977	852.4873	820.0001		

*t* statistics in parentheses.

* *p* < 0.1, ** *p* < 0.05, *** *p* < 0.01.

To validate the robustness of the preliminary test model and eliminate the potential impact of variables at different levels, we sequentially add different sets of explanatory variables to the regressions. The first column presents the results obtained by including only the basic firm level variables, while the second column displays the results of adding corporate governance variables. The third column represents the results of incorporating corporate openness variables, the fourth column displays the results of incorporating firm life cycle-related variables, and the last column shows the results of incorporating industry competition variables. An analysis of the signs and significance levels of the regression coefficients in each column indicates that the signs and significance levels of the coefficients remain unchanged and statistically significant after the progressive addition of various influencing factors, thus providing initial validation of the reliability of the model's findings.

The results of the regression analysis reveal that the coefficient for firm size is positive and significant at the 1% level, providing evidence for a positive correlation between firm size and the extent of a company's digital transformation, thereby supporting Hypothesis H1. This aligns with economic theory, which postulates that larger firms are more likely to have a higher degree of readiness and to undergo a greater extent of digital transformation. The larger a company's size, the more pressing the need to improve operational and management efficiency through digital transformation. This supports the credibility of Hypothesis H1.

The estimated coefficient of the gearing variable, which represents the capital structure, shows a significant positive relationship (at the 1% significance level) with the degree of digital transformation of A-share listed companies in the sample. This supports the hypothesis that higher gearing drives firms towards digital transformation, as companies with higher leverage seek to improve capital management efficiency and reduce the cost of capital through digital transformation. The same applies to the estimated coefficient of the accounts receivable percentage, which also has a significant positive relationship (at the 1% significance level) with the degree of digital transformation. This suggests that a higher percentage of accounts receivable prompts firms to deepen their digital transformation to improve their capital turnover and reduce their accounts receivable percentage. These results align with the logic of hypotheses H2 and H3, providing empirical evidence to support them.

The results of the regression analysis indicate that the coefficient for the overhead ratio (i.e. Management costs in Table 7) is positively significant at the 10% level, implying that enterprises with higher overhead costs are more motivated to undergo digital transformation with the aim of reducing expenses. These findings provide evidence to support Hypothesis H4, which posits that companies with higher overhead costs are more likely to increase their digital transformation efforts. Additionally, the regression results suggest that the effect of return on equity on digital transformation is negative, but not statistically significant, implying that return on equity is not a significant factor influencing digital transformation. This result supports the robustness of Hypothesis H5, which holds that return on equity is not a major determinant of digital transformation.

The results of the empirical study show that the equity concentration variable (i.e. Shareholding structure in Table 7), as a measure of corporate governance, has a significant negative correlation with the level of digital transformation, as indicated by its estimated coefficient, which is negative and significant at the 1% level. This supports hypothesis H7, which posits a negative relationship between equity concentration and the level of digital transformation. Furthermore, the heterogeneity test that we used to analyse the attributes of firms, such as duality, the share of overseas business, and the firm's life cycle, suggests that the level of digital transformation is influenced by the nature, characteristics, and stage of the firm, thus providing evidence in support of hypotheses H6, H8, H9, and H10.

In conclusion, the empirical results demonstrate that firms with higher debt ratios, higher accounts receivable ratios, and higher overhead costs are more likely to increase their digital transformation efforts in order to improve their operational efficiency and reduce costs.

### Robustness tests

In order to further verify the reliability of the empirical findings of this paper, robustness tests will be conducted using different methods.

#### Removal of exogenous interference

The COVID-19 pandemic in 2020 had a significant impact on the A-share listed companies, leading to a dual-faceted effect. On one hand, companies in a state of crisis may face difficulties in acquiring the financial and material resources required for digital transformation due to stagnant production and operations and disrupted cash flows. On the other hand, the pandemic lockdown prompted many companies to adopt digital transformation as a means of self-help, with some companies finding opportunities in the crisis to resume production to the greatest extent possible. As a result, in this study we exclude the observations from 2020 and 2021 to examine if the impact of each factor on the extent of digital transformation is in line with the regression results from the baseline model, which is not affected by exogenous shocks (Table 11).

**Table 11:** Robustness tests—excluding exogenous disturbances.

	(1) ln_digi2	(2) ln_digi2	(3) ln_digi2	(4) ln_digi2	(5) ln_digi2
Size	0.2368*** (27.98)	0.2440*** (28.73)	0.2492*** (29.28)	0.2494*** (29.08)	0.2509*** (29.25)
Lev	0.0125*** (3.15)	0.0139*** (3.51)	0.0142*** (3.58)	0.0133*** (3.36)	0.0137*** (3.46)
REC	0.7770*** (8.89)	0.7740*** (8.87)	0.8017*** (9.17)	0.7882*** (9.02)	0.7824*** (8.95)
ROA	0.0024 (0.10)	0.0053 (0.23)	0.0035 (0.16)	0.0054 (0.24)	0.0066 (0.29)
Mfee	0.0006** (2.41)	0.0005** (2.02)	0.0005** (2.04)	0.0006** (2.13)	0.0005** (2.05)
Top1		− 0.6655*** (− 10.11)	− 0.6585*** (− 10.01)	− 0.6140*** (− 9.24)	− 0.6073*** (− 9.14)
SOE		− 0.0302 (− 1.03)	− 0.0349 (− 1.19)	− 0.0390 (− 1.33)	− 0.0387 (− 1.32)
overseainc			− 0.2948*** (− 6.50)	− 0.2913*** (− 6.43)	− 0.2913*** (− 6.43)
ecycle				− 0.0521*** (− 2.77)	− 0.0512*** (− 2.72)
FirmAge				0.2162*** (4.31)	0.1875*** (3.72)
hhi_d					− 0.3072*** (− 5.63)
_cons	− 4.7855*** (− 26.57)	− 4.6771*** (− 25.97)	− 4.7512*** (− 26.33)	− 5.2216*** (− 24.89)	− 5.1474*** (− 24.48)
Year	Yes	Yes	Yes	Yes	Yes
*N*	29,786	29,786	29,773	29,773	29,759
adj. *R*^2^	0.273	0.276	0.277	0.278	0.278
F	862.0564	779.6973	744.9535	679.1195	651.0118

*t* statistics in parentheses.

* *p* < 0.1, ** *p* < 0.05, *** *p* < 0.01.

The robustness tests, performed with the exclusion of the potential impact of the COVID-19 epidemic on the digital transformation of enterprises, demonstrate the consistency of the results. Despite a slight reduction in the significance level of the firm attribute variable, the sign of the estimated coefficients of the other variables remained unchanged and statistically significant, indicating that the impact of the factors on the degree of digital transformation remains robust even in the absence of exogenous interference.

#### Substitution of explanatory variables

This subsection focuses on the exclusion of the term "digital technology applications" at the application level, and the retention of only the four basic digital technologies: "artificial intelligence technology", "blockchain technology", "cloud computing technology", and "big data technology". The frequency of occurrence of these four technologies is increased by 1, and the natural logarithm is used as a robustness test for the replacement variable. The results are as follows (Table 12).

**Table 12:** Robustness tests—replacement variables.

	(1) ln_digi	(2) ln_digi	(3) ln_digi	(4) ln_digi	(5) ln_digi
Size	0.2144*** (31.72)	0.2205*** (32.64)	0.2239*** (33.07)	0.2226*** (32.64)	0.2241*** (32.86)
Lev	0.0087*** (3.37)	0.0099*** (3.83)	0.0100*** (3.87)	0.0096*** (3.71)	0.0100*** (3.88)
REC	0.6333*** (9.35)	0.6377*** (9.45)	0.6482*** (9.59)	0.6417*** (9.49)	0.6398*** (9.47)
ROA	− 0.0091 (− 0.49)	− 0.0023 (− 0.12)	− 0.0035 (− 0.19)	− 0.0022 (− 0.12)	− 0.0007 (− 0.04)
Mfee	0.0001 (0.47)	0.0000 (0.20)	0.0000 (0.20)	0.0000 (0.24)	0.0000 (0.24)
Top1		− 0.6831*** (− 13.20)	− 0.6812*** (− 13.17)	− 0.6367*** (− 12.15)	− 0.6297*** (− 12.02)
SOE		0.0122 (0.56)	0.0095	0.0055 (0.25)	0.0060 (0.27)
			(0.43)		
overseainc			− 0.1231*** (− 3.43)	− 0.1213*** (− 3.38)	− 0.1242*** (− 3.47)
ecycle				− 0.0207 (− 1.36)	− 0.0198 (− 1.30)
FirmAge				0.2048*** (5.08)	0.1770*** (4.37)
hhi_d					− 0.3227*** (− 6.97)
_cons	− 4.4181*** (− 30.79)	− 4.2998*** (− 30.03)	− 4.3554*** (-30.36)	− 4.7929*** (− 28.69)	− 4.7217*** (− 28.21)
Year	Yes	Yes	Yes	Yes	Yes
*N*	37,823	37,823	37,823	37,823	37,823
adj. *R*^2^	0.231	0.236	0.237	0.238	0.239
F	832.3759	767.8006	734.8944	675.3626	650.4932

*t* statistics in parentheses.

* *p* < 0.1, ** *p* < 0.05, *** *p* < 0.01.

After adjusting the variables, the results of the regression are largely consistent with the benchmark regression, with the exception of a lower significance level of the overhead percentage, firm attribute, and life cycle variables (the direction of their estimated coefficients remain unchanged). This further illustrates the robustness of the empirical findings.

### Heterogeneity analysis

We further examine the impact of firm heterogeneity on the degree of digital transformation. The heterogeneity is evaluated in terms of enterprise attributes (state-owned enterprises vs. private enterprises), enterprise duality (whether the director and general manager are held by the same person), enterprise industry characteristics (whether it is a manufacturing industry), and enterprise life cycle (whether it is a recession). We also compare the differences and similarities in the effect of various factors on digital transformation for different types of firms (Table 13).

**Table 13:** Heterogeneity test results.

	(State enterprises) ln_digi2	(Private enterprises) ln_digi2	(Identical) ln_digi2	(Non-identical) ln_digi2	(Manufacturing) ln_digi2	(Other) ln_digi2	(Recession) ln_digi2	(The rest) ln_digi2
Size	0.1802*** (14.75)	0.2716*** (26.01)	0.2370*** (12.93)	0.2387*** (26.88)	0.1171*** (4.71)	0.2558*** (31.46)	0.2565*** (31.39)	0.2100*** (6.82)
Lev	0.0027 (0.06)	0.0093*** (2.95)	0.0043 (0.38)	0.0073** (2.38)	0.0104 (1.19)	0.0079** (2.49)	0.0107*** (3.20)	0.0020 (0.20)
REC	0.6986*** (5.53)	0.6374*** (6.64)	0.6046*** (3.85)	0.7923*** (8.91)	0.4448** (2.08)	0.7224*** (8.94)	0.7261*** (9.04)	0.8232*** (2.97)
ROA	0.2857*** (3.11)	− 0.0090 (− 0.41)	− 0.0044 (− 0.13)	− 0.0050 (− 0.17)	0.0054 (0.17)	− 0.0011 (− 0.04)	0.0490 (1.14)	− 0.0112 (− 0.39)
Mfee	0.0035 (0.49)	0.0003* (1.79)	0.0001 (0.12)	0.0002 (1.19)	0.0000 (0.05)	0.0003 (1.53)	0.0004** (2.19)	− 0.0011 (− 0.09)
Top1	− 0.3807*** (− 4.37)	− 0.6183*** (− 7.52)	− 0.7612*** (− 4.96)	− 0.5166*** (− 7.89)	− 0.9980*** (− 5.39)	− 0.6221*** (− 9.95)	− 0.6550*** (− 10.82)	− 0.3876 (− 1.26)
overseainc	− 0.0875 (− 1.27)	− 0.2756*** (− 5.63)	− 0.3993*** (− 5.05)	− 0.1708*** (− 3.54)	0.0713 (0.69)	− 0.2390*** (− 5.44)	− 0.2398*** (− 5.79)	− 0.3301* (− 1.75)
ecycle	− 0.0051 (− 0.21)	− 0.0283 (− 1.24)	0.0310 (0.88)	− 0.0287 (− 1.49)	− 0.0858* (− 1.90)	− 0.0139 (− 0.77)		
FirmAge	− 0.4341*** (− 6.09)	0.2022*** (3.42)	0.1710* (1.70)	− 0.0416 (− 0.78)	− 0.0610 (− 0.47)	0.1821*** (3.80)	0.0901** (1.96)	0.2063 (0.48)
hhi_d	− 0.1759** (− 2.41)	− 0.3796*** (− 5.38)	− 0.2476** (− 2.15)	− 0.3296*** (− 5.68)	− 0.6827* (− 1.87)	− 0.3135*** (− 5.93)	− 0.2979*** (− 5.58)	0.2232 (0.82)
SOE			− 0.1114 (− 1.48)	− 0.0029 (− 0.11)	− 0.1933*** (− 3.22)	0.0001 (0.00)	− 0.0603** (− 2.32)	− 0.0084 (− 0.08)
_cons	− 2.5261*** (− 8.18)	− 5.5315*** (− 22.14)	− 4.7979*** (− 11.22)	− 4.4738*** (− 20.23)	− 1.8028*** (− 3.12)	− 5.1871*** (− 25.98)	− 5.0405*** (− 25.67)	− 4.4090*** (− 3.39)
Year	Yes	Yes	Yes	Yes	Yes	Yes	Yes	Yes
*N*	14,169	23,654	10,312	27,511	5,319	32,504	35,608	2,215
adj. *R*^2^	0.293	0.288	0.190	0.279	0.206	0.300	0.296	− 0.395
F	301.5416	540.4887	197.6344	562.0322	87.9027	709.5102	806.6601	20.4412

*t* statistics in parentheses.

* *p* < 0.1, ** *p* < 0.05, *** *p* < 0.01.

#### Dimensions of firm attribute heterogeneity

1. In this research we have found significant differences in the influence of corporate capital structure and overhead ratios on the extent of digital transformation among firms of different attributes. The study has shown that there is a positive correlation between the degree of gearing and the degree of digital transformation in private firms. However, this relationship does not exist in state-owned enterprises as they can receive financial support from financial institutions and major shareholders in times of financial strain. In contrast, private firms often face challenges in obtaining financing and have high overhead costs, making digital transformation more important for them so as to reduce costs and minimize debt costs.
2. The impact of profitability on the extent of digital transformation is significant in state-owned enterprises but not in private companies. This can be attributed to the different perspectives of digital transformation held by the two types of firms. While private companies view digital transformation as a means to improve their performance, state-owned enterprises may see it as a way to enhance their already established success. The managerial mindset of state-owned enterprises, which is often influenced by prior experience in government work, inclines towards a more cautious and risk-averse approach, contributing to this difference in perception.
3. The analysis of both the entire sample and the private sample showed that there is a positive correlation between a firm's age and the degree of its digital transformation. This result is consistent with the difference in profitability between the two samples, reflecting the overall cautious approach of state-owned enterprises compared to private firms in terms of the factors impacting digital transformation.

#### Dimensions of corporate duality

In our examination of the effect of corporate duality on the level of digital transformation, we found a notable distinction in the impact of the firm's age. For firms where the Chairman and the General Manager are the same individual, there is a positive correlation between the firm's age and the level of digital transformation, meaning that as the firm grows older, the level of digital transformation increases. Conversely, for firms where the Chairman and General Manager are different individuals, there is no statistically significant association between the firm's age and the degree of digital transformation. This discrepancy may be due to information asymmetry between the principal and agent in the latter type of firms, leading to a lack of transfer of operational experience from one General Manager to the next and causing a generation gap in management as the company ages.

#### Dimensions of industry heterogeneity

1. For the manufacturing sector, there is no statistically significant correlation between a firm's gearing and the degree of digital transformation. Conversely, in non-manufacturing industries, a higher gearing ratio was found to correspond with a higher degree of digital transformation. This disparity can be attributed to the fact that manufacturing companies typically have high gearing ratios, while non-manufacturing companies are more asset-light and may find it more challenging to secure financing, leading to a greater need to reduce their debt burden through digital transformation.
2. The results of the analysis showed that the relationship between the degree of digital transformation and the percentage of overseas business revenue differs between manufacturing and non-manufacturing companies. In the case of non-manufacturing firms, there is a negative correlation between the percentage of overseas business revenue and the degree of digital transformation, whereas for manufacturing firms, there is no significant association between the two variables. Additionally, the results indicated that for manufacturing firms, the degree of digital transformation is highest during the maturity stage of the life cycle, whereas for non-manufacturing firms, there is no significant difference in the degree of digital transformation among companies in different life cycle stages.

#### Dimensions of life cycle heterogeneity

1. We found that companies experiencing recession tend to adopt digital transformation more intensively to decrease their gearing. This relationship between gearing and digital transformation was not found for companies at other stages of their life cycle. This can be attributed to the difficulty faced by companies in recession in obtaining external financing, leading them to optimize their operations and create new revenue streams through digital transformation to reduce their debt. The same phenomenon was observed with respect to overhead ratios, with non-recessionary companies being more tolerant of high overhead costs due to the characteristics of their operations prior to the recession.
2. For firms that are not in recession, no significant correlation was found between the degree of digital transformation and equity structure, company age, and industry competition, which are factors that usually have a major impact. This can be attributed to the need for companies to have a clear understanding of the factors affecting their digital transformation during the initial and growth stages. Management philosophies may vary, leading to diverse decision-making processes and a lack of uniformity in comparison to companies in the declining stage.

## Discussion

### Research findings

This study has uncovered several key insights into the influencing factors of corporate digital transformation among Chinese listed companies. Firstly, the gearing ratio analysis illustrates that digital transformation facilitates optimised asset allocation, reducing gearing ratios and enhancing asset utilisation efficiency. JD.com is a prime example, where digital technologies streamlined inventory and supply chain management, reducing the gearing ratio and improving asset turnover^75^. The inclination towards digital transformation in companies with higher gearing ratios can be attributed to the pressure to enhance financial stability and reduce dependency on borrowed capital. Companies aim to attract better investment opportunities and mitigate financial risks by optimising assets and reducing liabilities.

Secondly, adopting automation and intelligent technology significantly decreases management expenses regarding the management overhead ratio. This results in improved efficiency and reduced workforce costs, boosting overall competitiveness. Xiaomi Inc.'s integration of AI into its operations exemplifies how digital transformation can lead to operational efficiencies and cost reductions^76^. Companies with high overhead ratios pursue digital transformation to streamline operations and reduce costs, driven by the need to enhance profitability and competitiveness in a fast-evolving market.

Finally, from the perspective of accounts receivable ratio, digital transformation can help enterprises optimise accounts receivable management, accelerate the speed of capital turnover, reduce the risk of bad debts, and improve the liquidity and solvency of enterprises. Alibaba Group's use of blockchain technology for accounts receivable processes exemplifies the potential of digital solutions to enhance financial operations^77^. The motivation here stems from the desire to accelerate capital turnover and improve financial health, particularly for companies facing high accounts receivable ratios that can hinder cash flow and increase financial vulnerability. Therefore, companies with high gearing, overhead, and accounts receivable ratios are more likely to drive digital transformation to minimize costs and enhance performance.

However, companies in monopolistic industries and those in the inception stage of their life cycle are less likely to undertake significant digital transformation. This cautiousness is attributed to the potential risks of disrupting established processes and the significant investments required for major digital overhauls. Instead, they may adopt a more cautious approach and make incremental changes to avoid disrupting the existing business environment.

This research reinforces the observations by Ferreira, Fernandes and Ferreira^68^ and Chanias, Myers and Hess^57^, who highlighted the role of digital transformation in optimizing asset allocation and reducing management expenses in European and North American firms, respectively. However, we extend these insights by demonstrating the pronounced effect of digital initiatives in enhancing financial stability and reducing the gearing ratio. Digital transformation can help enterprises optimise asset allocation, reduce gearing ratio and improve asset utilisation efficiency, thus creating more value for enterprises. Also, in terms of management expense ratio, digital transformation can reduce the management expense ratio of the enterprise through automation and intelligent technology, improve management efficiency, reduce manpower costs and improve the overall competitiveness of the enterprise.

Furthermore, this study enriches the discourse initiated by Strange, Chen and Fleury^61^ on the benefits of digital transformation in the manufacturing industries by exploring its impact on the Chinese economy. It uncovers that digital practices significantly expedite capital turnover and diminish debt risks, improving liquidity and solvency in an acutely relevant manner for Chinese firms. From the perspective of accounts receivable ratio, digital transformation can help enterprises optimise accounts receivable management, accelerate the speed of capital turnover, reduce the risk of bad debts, and improve the liquidity and solvency of enterprises.

Rooted in existing research, the main contributions of this paper are altogether as follows: first, to identify the factors that influence the intensity of digital transformation of firms, including both internal firm factors and external industry factors. Although previous studies have examined the economic impact of digital transformation, the research in this paper is able to explain in more detail the reasons for the differences in the degree of digital transformation among different firms; second, this paper incorporates enterprise micro characteristics and industry factors into the analysis, examining the heterogeneity of enterprise attributes, enterprise duality, industry characteristics and enterprise life cycle, so as to propose targeted policy recommendations; third. The complexity of the enterprise digital transformation process makes it difficult to measure the degree of transformation, and relevant statistics are relatively limited. This research further complements and enriches the index portrayal of digital transformation by measuring it through regression models with the help of annual reports of A-share listed companies.

### Recommendations

1. Policymakers: Offer public and platform assistance in science and technology policies to facilitate the extensive utilization of digital advancements like big data, artificial intelligence, and cloud computing in all facets of business activities such as research and development, design, production, operation, and sales management. Bai et al.^30^ suggest that government should enhance funding for capital, workforce, and technological innovation initiatives to cater to the public and quasi-public service requirements of enterprises, particularly small and medium-sized businesses (SMEs), in order to facilitate their digital transition, including the establishment of industry-wide digital technology platforms.
2. Business leaders: To navigate the complexities of digital transformation successfully, Chinese listed companies must adopt a strategic approach that aligns with their unique operational and market contexts. As Osmundsen, Iden and Bygstad^78^ emphasize, the path to a digital ecosystem necessitates a tailored strategy that considers the specific needs and challenges of each company, ensuring that digital transformation initiatives are not just adopted for their own sake but are integrated in ways that genuinely enhance organizational performance and competitive advantage. Hence, enterprises should selectively carry out digital transformation according to their own situation and development needs. Before embarking on digital transformation, the management should make adequate preliminary preparations such as conducting an internal review of the enterprise, getting a full grasp of the enterprise's development, and setting a clear corporate development strategy and long-term goals, to understand the situation of the enterprise itself; subsequently they should engage in such exercises as market research to gain an in-depth understanding of market demand, as well as consumer preferences, and so on, to understand the external environment of the enterprise^79^.
3. Other stakeholders: Stakeholders including relevant practitioners, employees should cultivate digital thinking and receive digital training. Cultivating practitioners' digital thinking is beneficial to strengthening their understanding of digital transformation, which helps them make the right decisions and is conducive to the survival and development of enterprises. Digital thinking includes building a digital vision, grasping the direction of transformation, maintaining strategic strength and other dimensions; enterprises should use digital thinking to solve practical problems in the process of enterprise development^80^. Meanwhile, navigating and leveraging government policies and incentives for digital transformation is crucial for maximizing the benefits of these initiatives. Verhoef et al.^70^ provide a roadmap for Chinese enterprises to effectively engage with government-led digital ecosystems and take advantage of policy support, underscoring the strategic importance of public-private partnerships in facilitating digital innovation. By actively participating in these ecosystems, companies can access a broader network of resources, technologies, and market opportunities essential for successful digital transformation.

### Conclusion and outlook

This article focuses on the influencing factors of the degree of digital transformation of enterprises, and selects the important influencing factors at the levels of enterprise characteristics, corporate governance, enterprise internationalization, enterprise life cycle, and industry competitiveness, and explores the influence of these factors on the degree of digital transformation of enterprises. The empirical results also reflect the motivation of enterprises' digital transformation, which seems to be more of a "sending charcoal in the snow" motivation than an "adding flowers on the cake" motivation in this paper's sample observation range.

The paper concludes with countermeasures for enterprise digital transformation from the enterprise and government levels. Enterprises need to carry out digital transformation according to their own situation and needs, and make full use of top-level design and market research. Enterprise practitioners should have a deep understanding of the connotation of digital culture, and strengthen the construction of the digital talent team. At the government level, investment in digital infrastructure should be increased to ensure its viability and receive political and financial support.

One limitation of this research is its insufficient coverage of the technological aspect within the TOE framework. Given the significant role that technology plays in today's business landscape, overlooking this perspective could hinder the comprehension of how the TOE framework is implemented within an organization. The focus of this study is primarily on the influence of organizations and environments on technology adoption and usage, rather than delving into the technology itself. This lack of emphasis on technology may result in readers not fully grasping the practical applications and impacts of technology on the TOE framework. Neglecting to properly address the technological aspect may lead to an incomplete understanding of how corporate decisions regarding technology adoption and utilization are made, potentially resulting in biased or erroneous conclusions in real-world scenarios.

To gain a more comprehensive understanding of how the TOE framework operates in practice, future research endeavors should integrate the technical viewpoint to offer a more well-rounded perspective on technology adoption. We intend to manually gather pertinent data in order to provide a thorough and detailed examination of the subject matter.

## Data Availability

The data that support the findings of this study are available from the corresponding author upon reasonable request.
